# Tryptophan Depletion Promotes Habitual over Goal-Directed Control of Appetitive Responding in Humans

**DOI:** 10.1093/ijnp/pyv013

**Published:** 2015-02-06

**Authors:** Yulia Worbe, George Savulich, Sanne de Wit, Emilio Fernandez-Egea, Trevor W. Robbins

**Affiliations:** Behavioral and Clinical Neuroscience Institute (Drs Worbe, Fernandez-Egea, and Robbins), Department of Psychiatry (Drs Savulich and Fernandez-Egea), and Department of Psychology (Dr Robbins), University of Cambridge, Cambridge, United Kingdom; Department of Clinical Psychology, University of Amsterdam, Amsterdam, The Netherlands (Dr de Wit).

**Keywords:** serotonin, 5-HT, goal-directed behavior, stimulus-response habit learning, inhibitory control, tryptophan depletion

## Abstract

**Background:**

Optimal behavioral performance results from a balance between goal-directed and habitual systems of behavioral control, which are modulated by ascending monoaminergic projections. While the role of the dopaminergic system in behavioral control has been recently addressed, the extent to which changes in global serotonin neurotransmission could influence these 2 systems is still poorly understood.

**Methods:**

We employed the dietary acute tryptophan depletion procedure to reduce serotonin neurotransmission in 18 healthy volunteers and 18 matched controls. We used a 3-stage instrumental learning paradigm that includes an initial instrumental learning stage, a subsequent outcome-devaluation test, and a slip-of-action stage, which directly tests the balance between hypothetical goal-directed and habitual systems. We also employed a separate response inhibition control test to assess the behavioral specificity of the results.

**Results:**

Acute tryptophan depletion produced a shift of behavioral performance towards habitual responding as indexed by performance on the slip-of-action test. Moreover, greater habitual responding in the acute tryptophan depletion group was predicted by a steeper decline in plasma tryptophan levels. In contrast, acute tryptophan depletion left intact the ability to use discriminative stimuli to guide instrumental choice as indexed by the instrumental learning stage and did not impair inhibitory response control.

**Conclusions:**

The major implication of this study is that serotonin modulates the balance between goal-directed and stimulus-response habitual systems of behavioral control. Our findings thus imply that diminished serotonin neurotransmission shifts behavioral control towards habitual responding.

## Introduction

In humans and other animals, optimal behavioral performance results from a balance between adaptive flexible control and more rigid, repetitive choices, which are supported by goal-directed and habitual systems, respectively (Everitt and Robbins, 2005b; Graybiel, 2008; Balleine and O’Doherty, 2010). Flexible behaviors are adaptive for attaining desirable goals and require knowledge of the consequences of one’s instrumental behavior through stimulus-outcome-reward associations. In contrast, cognitively less demanding fixed routines may be advantageous in certain situations, for example, under stress. These habitual behaviors are usually driven by contextual cues through stimulus-response (S-R) associations and are insensitive to goal value. Consequently, a classic test for the existence of S-R control is to observe an absence of effect of goal devaluation (eg, via satiation) on behavior (Dickinson, 1985; Balleine and O’Doherty, 2010).

Goal-directed and habitual systems may act synergistically or competitively and their abnormal interactions could potentially lead to neuropsychiatric disorders. For instance, abnormal habit formation has been suggested to play a key role in disorders such as substance addiction (Everitt and Robbins, 2005a; Sjoerds et al., 2013) or obsessive-compulsive disorder (OCD) (Gillan et al., 2011).

For normal behavioral control, ascending monoaminergic projections modulate the balance between the 2 systems. In particular, dopamine (DA) has been shown to play a role in both habitual and goal-directed behaviors. Thus, in rodents, chronic amphetamine administration resulting in behavioral sensitization (probably via enhanced DA neurotransmission) resulted in acceleration of habit formation (Nelson and Killcross, 2006), which was reversed by a D1 DA receptor antagonist (Nelson and Killcross, 2012). In contrast, lesions of the nigro-striatal DA pathways in rodents disrupted habit formation (Faure et al., 2005).

Another monoamine neurotransmitter, serotonin (5-hydroxytryptamine [5-HT]), is considered to be in both an opponent and a synergistic functional relationship with brain DA, but its precise role in decision-making is still unclear. Facilitation of behavioral flexibility has been associated with the overexpression of striatal serotonin receptors type 6 (5-HT_6_) receptors in the striatum (Eskenazi and Neumaier, 2011), whereas perseveration during reversal learning occurred in monkeys with selective prefrontal serotonin depletion (Clarke et al., 2004). Moreover, certain forms of compulsive behavior, such as persistent cocaine-seeking in rats, are associated with reduced forebrain serotonin levels (Pelloux et al., 2012). Finally, OCD is generally treated chronically with high doses of selective serotonin reuptake inhibitors, which presumably boost 5-HT transmission.

Consequently, in this study we aimed to test the hypothesis that central serotonin depletion would alter the balance between goal-directed and habitual responding by shifting the balance towards the latter. To address this hypothesis, we employed the dietary acute tryptophan depletion procedure (ATD) to reduce 5-HT neurotransmission in healthy volunteers. This procedure has been shown to produce a transient reduction of central serotonin transmission in the brain, as tryptophan is the amino acid precursor of serotonin (Biggio et al., 1974; Carpenter et al., 1998; Ardis et al., 2009). Moreover, previous studies showed similar behavioral effects induced by ATD in healthy volunteers compared with the selective serotonin lesions in animal models (Worbe et al., 2014). In addition, both animal and human studies have suggested a selective ATD effect on central serotonin, with no effect on DA and norepinephrine neurotransmission (Ardis et al., 2009; Cox et al., 2011).

**Figure 1.**
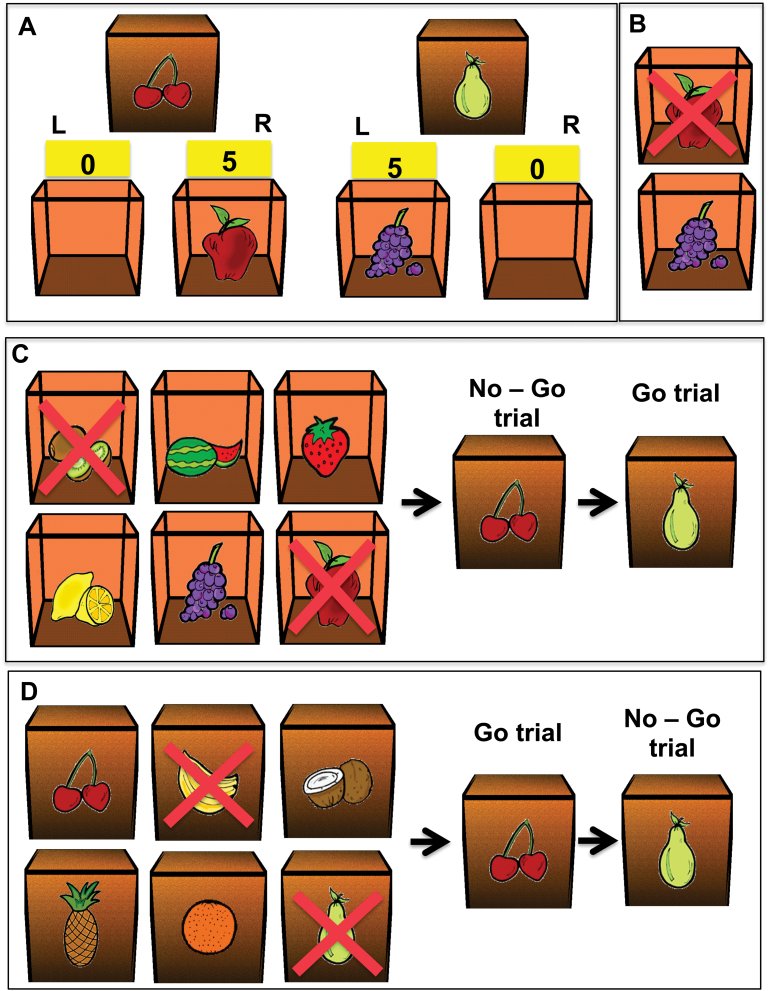
Instrumental learning task. (A) Instrumental learning stage. In this example, in 2 different trials, participants were presented with cherries and pear on the outside of the box. For each of the trials, if the correct key was pressed (R – right for cherries and L – left for pear), participants were rewarded with another fruit (apple and grapes, respectively) on the inside of the box and points. If the incorrect key was pressed, an empty box was shown and no points were earned. (B) Outcome-devaluation test. In this example, 2 open boxes with an apple and grapes inside were presented to the participants. The fruit (the apple in this example), which was no longer worth any points, was indicated with a red cross on it. The participant should press the correct key for the still-valuable fruit (left key in this example for the still-valuable grapes symbol). (C) Slips-of-action test. In this example, the initial instruction screen showed that the kiwi and the apple outcomes (indicated by the red crosses) will now lead to the subtraction of points. The other 4 outcomes were still valuable. Following the instruction, participants were shown in rapid (2 seconds) succession the fruit stimuli (on the front doors of the boxes) and were asked to press the correct keys (Go-trial) for the still-valuable outcome stimuli (inside the box) but to refrain from responding (No-Go trials) when the outcome stimuli were devalued. In this particular example, participants should withhold responding when the cherry stimulus was shown but press the left key for the pear stimulus. (D) Baseline control test of inhibitory response. In this example, the initial instruction screen showed that 2 of the cueing stimuli on the outside of the boxes indicated by the red crosses (banana and pear) would now lead to the subtraction of points. The other 4 stimuli were still valid. Following the instruction, participants were shown the fruit stimuli in rapid (2 seconds) succession and asked to press the correct keys (Go-trial) for the still-valid stimuli but to refrain from responding (No-Go trials) when the stimuli were devalued. In this particular example, participants should withhold response when the pear stimulus was shown but press the right key for the cherry stimulus.

We used a 3-stage instrumental learning paradigm (de Wit et al., 2007), which includes an initial instrumental learning stage, where participants learned by trial and error which responses (right or left key presses) were cued as being correct by different pictures of fruit that served as discriminative stimuli (Figure 1A-D). Correct responses were always rewarded by other fruit pictures and their associated points (which could ultimately be exchanged for money). In a subsequent outcome-devaluation test (when certain fruits no longer earned points), healthy volunteers had to use their knowledge of the response-outcome (R-O) associations to direct their choices towards still-valuable fruit outcomes and away from no-longer-valuable outcomes. In the last stage of the paradigm, the balance between goal-directed and habitual systems was directly tested as participants were asked to selectively respond to stimuli that signaled the availability of still-valuable outcomes, whereas they were still required to withhold responding to stimuli that signaled devalued outcomes. We also included an internal control test for possible impairments in the general capacity of response (or motor) inhibition. The slips-of-action test was previously shown to be sufficiently sensitive to evaluate the balance between goal-directed and habitual behavioral control both in pathological conditions (OCD) (Gillan et al., 2011) and following dietary manipulation of tyrosine, a precursor of the neurotransmitter DA (de Wit et al., 2012b). The latter study found that dietary tyrosine depletion greatly enhanced habit learning in female volunteers, consistent with a role for DA in S-R habit learning.

Based on the considerations above, we hypothesized that ATD would selectively enhance habitual response control while leaving intact the capacity of subjects to learn from the instrumental stage of the paradigm.

## Materials and methods

### Participant Inclusion Criteria

The East of England-Essex Research Ethics Committee approved the study. Participants were recruited from university-based advertisements and from the Cambridge BioResource (www.cambridgebioresource.org.uk) and gave informed consent prior to the study. The inclusion criteria were age between 18 and 45 years and absence of history of neurological, psychiatric (assessed with the Mini International Neuropsychiatric Inventory; Sheehan et al., 1998) or any significant physical illness, absence of drug abuse including nicotine, and not currently taking any type of regular medication (except contraceptive pills for women).

### Experimental Procedure

Participants were assigned to receive either the acute tryptophan-depleting drink (ATD) or the placebo (BAL) mixture in a randomized, placebo-controlled, double-blind order. They were asked to abstain from food and alcohol 12 hours prior to the testing session.

In the ATD procedure, tryptophan was depleted by ingestion of a liquid amino acid load that did not contain tryptophan but did include other large neutral amino acids (LNAAs). The biochemical composition of drinks can be found in the supplementary Materials. We did not perform the adjustment of the drink’s composition for the female participants. The participants were informed about potential adverse effects of drink ingestion, but none of them reported the adverse effects such as nausea or vomiting.

At baseline, the first blood sample was taken and we administrated the Beck Depression Inventory (Beck et al., 1996), the Spielberg State-Trait Anxiety Inventory (STAI) (Spielberger, 1989), and the Barratt Impulsivity Scale (Patton et al., 1995). The STAI-state were also administered at additional time points of the study. A proxy measure of intelligence quotient was the National Adult Reading Test (Bright et al., 2002). Behavioral testing was performed and the second blood sample taken after a resting period of approximately 4.5 hours to ensure stable and low tryptophan levels.

### Biochemical Analysis

Immediately after venepuncture, blood (7mL) was centrifuged at 2000rpm for 20 minutes, and serum was separated by centrifugation and stored at -80°C. Plasma total amino acid concentrations (tyrosine, valine, phenylalanine, isoleucine, leucine, and tryptophan) were measured by means of high-performance liquid chromatography with fluorescence end-point detection and precolumn sample derivatization. The tryptophan/large neutral amino acid (TRP:ΣLNAAs) ratio was calculated as an indicator of central serotoninergic function (Carpenter et al., 1998).

### Instrumental Learning Task

We used an adapted version of the instrumental learning task (de Wit et al., 2007) that was programmed in Visual Basic 6.0 and was divided into 3 stages: instrumental learning, outcome-devaluation test, and slips-of-action test. The mean duration of the task was 40 minutes.

### Instrumental Learning Stage

At the beginning of each trial, a closed box was shown on a computer screen with a picture of a fruit on the front. This symbol served as a discriminative stimulus, signalling that participants should press either a right or left key, one of which would be rewarded with another fruit (inside the box) and points (Figure 1A). Faster correct responses earned more points (from 1 to 5). Following incorrect responses, the box was empty and no points were earned. By trial and error, participants should figure out which key to press for 6 different fruit pictures on the outside of the box. They were instructed to earn as many points as they could and to pay attention to what fruit was inside the box. The stage was divided in 8 blocks of 12 trials each (96 trials in total). There were 6 stimuli-outcome associations. The main outcome measures on this task stage are the learning rate and the reaction time (RT).

### Outcome Devaluation Stage

The test was conducted to assess R-O knowledge. During each trial of the outcome devaluation stage (Figure 1B), 2 fruit outcomes were presented together: one outcome for a right response and one for a left response. The 6 possible combinations each appeared 6 times during this test stage. One of the fruits was shown with a cross on it to indicate that it was no longer rewarded by points (devalued). Participants were instructed to press the key that would allow them to collect the still-valuable fruit. The test consisted of 36 trials. No feedback on performance was provided during the test, but participants were told that they would earn a point for each correct response. The cumulative number of points was displayed after test completion. The accuracy is the main outcome measure of this stage.

### Slips-of-Action Stage

This test allowed direct assessment of relative habitual and goal-directed behavioral control. At the beginning of each block of trials (6 blocks in total), all 6 fruit outcomes from the initial training stage were shown on the screen (Figure 1C). The red cross on 2 of the fruits indicated that their collection would now lead to subtraction of points. Following this screen (10 seconds presentation), a series of closed boxes with the fruit stimuli on the front were presented in rapid succession (2 seconds per trial). Participants were instructed to press the appropriate keys to open boxes that contained still-valuable outcomes (go trials) to gain points. To avoid losing points, participants were to refrain from responding if a box contained a now-devalued fruit (No-Go trials). Each of the 6 stimuli was shown 4 times per block, and across blocks, each of the outcomes was devalued twice. Participants completed 144 trials in total, and they were shown the cumulative number of points at the end of the test.

**Figure 2.**
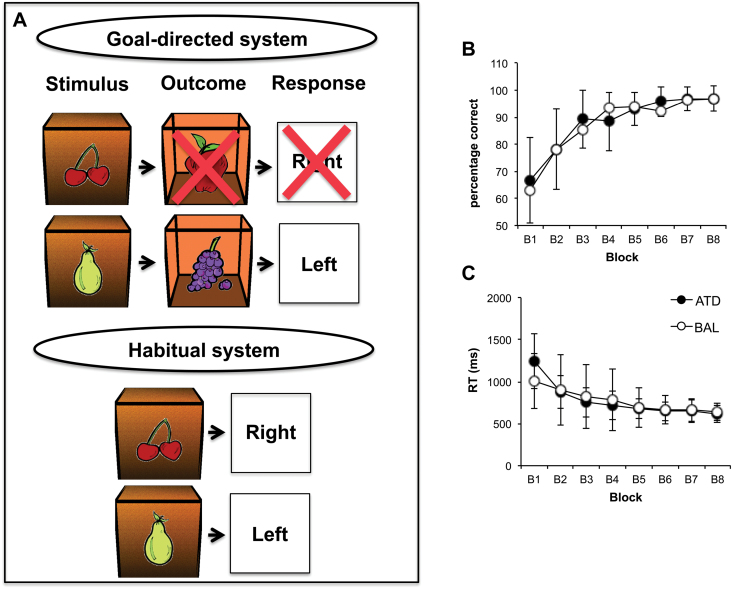
(A) Behavioral results in a slip-of-action test: the goal-directed system (stimulus-response-outcome association) allows a successful performance based on action outcome evaluation; the habitual system, based on stimulus-response (S-R) association, activates response directly and will enable slips of action toward no-longer-valuable outcomes. (B) Percentage of correct responses in the instrumental learning stage of the task. (B1-8) The testing blocks of 12 trials each (96 trials in total). (C) Reaction time to the key press in the instrumental learning stage.

As shown in Figure 2A, if the goal-directed system exerts dominant control over behavior, this should result in selective responding towards valuable as opposed to devalued outcomes. The dominance of the habitual control system should lead to commission errors on trials with the devalued outcomes. The main outcome measures are the percentage of responses and accuracy on trials where either valuable or devalued outcomes were available.

### Baseline Test of Response Inhibition

This additional test was randomly performed before or after slip-of-action test with the same number of trials and blocks. The baseline test was identical to the slips-of-action test, except that the discriminative stimuli were “devalued” instead of the outcomes. This essentially meant that participants had to decide to go or no-go on the basis of the stimuli that they were shown directly during the test phase. Therefore, unlike the slips-of-action test, performance on this test did not require anticipation and evaluation of the action outcomes. However, any general impairment in either response inhibition of working memory should impair performance equally on the 2 tests.

At the beginning of each block of trials (Figure 1D), pictures of 6 initial stimuli (closed boxes with a picture of a fruit on the front) were presented to the participants. A red cross on 2 of the fruits indicated that their collection were incorrect (No-Go) and would now lead to subtraction of points. Following this screen, the boxes were presented in rapid succession, and participants were instructed to press the appropriate keys to still correct boxes and withhold the response to the incorrect (No-Go) stimuli. The outcome measures of this test were percentage of responses towards correct (Go) versus incorrect (No-Go) stimuli RT, and response accuracy.

### Statistical Analysis

Statistical analysis was performed using Statistical Package for Social Science (SPSS) version 21 (SPSS Inc., Chicago, IL). Prior to analysis, all variables were tested for Gaussian distribution (Shapiro-Wilk test, *P*<.05; with square root transformation if necessary) and outliers data. Demographic data were analysed using 2-sample *t* test. The analyses of data from biochemical analyses were performed using mixed-measures ANOVA with time as a dependent factor and group as a fixed factor. Behavioral data were analyzed using a mixed-measure ANOVA with group as a fixed factor and the outcome measures of the instrumental learning task as dependent factors. Posthoc comparisons were performed using Tukey’s test.

## Results

### Subjects

Forty-two subjects were randomly allocated to the BAL or ATD groups. Three subjects in each group failed to satisfactorily complete the first stage of the task and were excluded from the final analysis of the data. The groups’ demographic data are reported in Table 1. There was no significant difference between groups in baseline impulsivity, mood or anxiety trait-state evaluation, and National Adult Reading Test scores. Four women in the BAL group and 5 in the ADT group used contraceptive pills. We did not control for menstrual cycle in female participants.

**Table 1.** Demographic and Behavioral Data of Participants

	**Controls** **(n = 18)**	**ATD group** **(n = 18)**	***F***	***P***
Age	26.59±1.50	26.07±3.68	0.23	.88
Men (n)	8	8		
IQ	121.15±2.16	121.50±1.84	0.65	.44
BDI	3.95±0.92	3.64±1.10	0.037	.83
STAI, trait	47.31±1.41	44.84±1.10	1.46	.23
STAI, state	41.77±1.44	43.69±1.36	0.87	.35
Barratt impulsivity scale	69.545±6.456	70.571±5.153	1.0	.32

Abbreviations: ATD, acute tryptophan depletion; BDI, Beck Depression Inventory; IQ, intelligence quotient; STAI, Spielberg State-Trait anxiety Inventory.

### Biochemical Measures

Tryptophan depletion robustly decreased the TRP:ΣLNAAs ratio relative to the BAL (main effect of group: F_(1,34)_ = 20.402, *P*<.0001; main effect of time: F_(1,34)_ =3.732, *P*=.042; group × time interaction: F_(1,34)_ = 16.342, *P<*.001). The mean (± SEM) baseline and postprocedure values of TRP:ΣLNAAs ratio were as follows: baseline – BAL (0.164±0.015), ATD -group (0.134±0.007); postprocedure - BAL (0.194±0.014), ATD group (0.060±0.020).

To control for mood and anxiety state, we administered the STAI-state questionnaire at baseline and at 4, 6, and 8 hours after drink intake. There was no significant effect of TD on anxiety state: main effect of time (F_(1,34)_ = 2.349, *P*>.05), main effect of group F_(1,34)_=2.579, *P*>.05). We also found no correlation of TRP:ΣLNAAs ration with anxiety state (all *P*>.05).

### Effect of Tryptophan Depletion on Instrumental Learning

Using a mixed-measure ANOVA, we first compared the learning rate in the instrumental learning stage of the task. As shown in Figure 2B, both groups significantly increased their performance over the test blocks (main effect of learning: F_(1,34)_=77.645, *P<*.0001, η^2^_p_=0.695), with no difference in performance between the groups (main effect of group: F_(1,34)_ < 1.0; with the mean percentage of correct response, mean±SEM, BAL: 86.590±1.697; ATD: 89.236±1.794).

Over the training blocks, the RT on the stimuli became significantly shorter in both groups (mean effect of block on RT: F_(1,34)_ = 59.975, *P<*.0001, η^2^=0.638), with no difference in the RT between groups (mean effect of group, F_(1,34)_ < 1.0; ms; mean ± SEM, BAL: 780±61; ATD: 760±32) (Figure 2C).

### Effect of Tryptophan Depletion on Outcome-Devaluation Test

Multivariate ANOVA with accuracy and RT as dependant factors and group as independent factor, a group effect on the RT (F_(1,34)_ = 7.751, *P*=.009, η^2^_p_ = 0.186), where the ATD group was significantly slower in responding to stimulus presentation (ms, mean±SEM, BAL: 1100.12±71.69; ATD: 1517.15±127.36) but with no effect on accuracy of performance (F_(1,34)_ = 2.83, *P* = .10; percent of accurate response, mean ± SEM, BAL: 93.98±1.75; ATD: 88.27±2.90).

### Effect of Tryptophan Depletion on Balance between Goal-Directed and Habitual Control on Slip-Of-Action Test

First, we performed an ANOVA analysis with test type (baseline, slip-of-action) and devaluation (valuable, devalued) as within-subject factors and group (ATD vs BAL) as between-subjects factor. This analysis revealed significant main effects of test (F_(1,34)_ =19.27, *P<*.001, η^2^_p_=0.362), devaluation (F_(1,34)_ = 290.69, *P<*.0001, η^2^_p_ = 0.895), and a test × devaluation × group interaction (F_(1,34)_ = 4.66, *P* = .038, η^2^_p_ = 0.683).

In further analysis of the slip-of-action test, mixed-measures ANOVA with group (ATD vs BAL) as an independent factor and devaluation (valuable and devalued) as dependent factor showed main effects of devaluation (F_(1,34)_ = 42.08, *P<*.001, η^2^_p_ = 0.553) and group (F_(1,34)_ = 3.62, *P* = .046, η^2^_p_ = 0.126) and a significant group × devaluation interaction (F_(1,34)_ = 5.31, *P* = .027, η^2^_p_ = 0.135). On posthoc comparison, the ATD group made significantly more responses for devalued stimuli compared with BAL (F_(1,34)_ =5.46, *P* = .025, η^2^_p_=0.138; percent or response, mean ± SEM, BAL: 35.18 ±5.41; ATD: 54.01±5.95), whereas there was no difference in responses for valuable outcomes (F_(1,34)_ < 1.0; percent or response, mean ± SEM, BAL: 81.22±2.34; ATD: 75.90±3.11).

Given that a response was made for a devalued outcome, there was no effect of ATD on response accuracy (F_(1,34)_ =2.42, *P*=.13) or RT (F_(1,34)_ <1). There was also no difference between the groups in response accuracy (F_(1,34)_ <1) for valuable outcomes (F_(1,34)_ =1.59, *P*=.23) or RT (F_(1,34)_ =1.5, *P*=.228; RT, ms, mean ± SEM, BAL: 1010.34±36.52; ATD: 1071.72±34.09).

**Figure 3.**
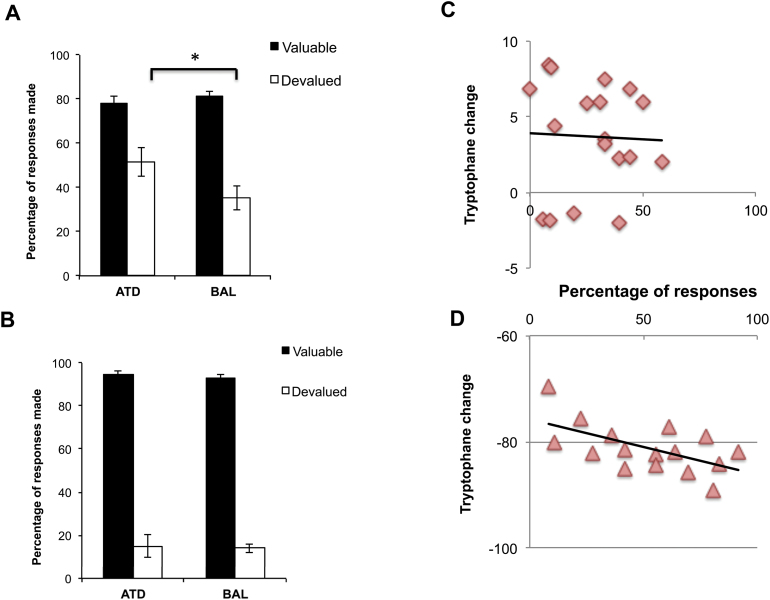
(A) Percentage of response on valuable and devalued stimuli in the slips-of-action test. (B) Percentage of response to correct (Go) and incorrect (NoGo) stimuli in the internal control test of inhibitory response control. (C-D) Regression analysis of percentage of responses to the devalued outcomes out of the total number of trials with devalued outcomes in slip-of-action test over percentage of tryptophan reduction in peripheral blood in acute tryptophan depletion (ATD) and control group (BAL) (C) and BAL (D).

Further, we specifically tested the hypothesis that level of tryptophan reduction would be correlated with the shift to habitual responding in the slip-of-action test. Linear regression of the percentage of responding for the devalued outcomes in the ATD group over the percentage of the tryptophan level reduction was calculated as a difference of pre- and postprocedure tryptophan levels in the peripheral blood. In the ATD group, the higher percentage of responses for devalued outcomes corresponded to the higher level of the tryptophan reduction in peripheral blood (β = -0.503, *P*=.045), whereas this effect was not found in the BAL croup (β=0.034, *P* = .894) (Figure 3C-D).

### Effect of Tryptophan Depletion on the Baseline Test of Response Disinhibition

In the control test, a mixed-measure ANOVA with group as an independent factor and devaluation (valuable and devalued) as a dependent factor showed a main effect of devaluation (F_(1,34)_ = 913.77, *P<*.0001, η^2^_p_ = 0.955), but no effect of group (F_(1,34)_ < 1) nor a group × devaluation interaction (F_(1,34)_ < 1). Posthoc comparisons showed no significant differences between the groups in response percentage and accuracy on trials with valuable and devalued stimuli (all F_(1,34)_ < 1). If responding for the devalued stimuli occurred, the ATD group had slower mean RT compared with the BAL (F_(1,34)_ = 4.62, *P* = .037, η^2^_p_ = 0.094; RT, ms, mean ± SEM, BAL: 837.57±64.04; ATD: 1057.27±80.22).

## Discussion

Using a 3-stage instrumental learning task, we showed a shift in balance from goal-directed to habitual responding in healthy volunteers submitted to the ATD procedure, which is presumed to cause reduced central 5-HT function (Crockett et al., 2012). The ATD group had a significantly higher level of responding on the slip-of-action test when the discriminative stimuli indicated that the outcome had been devalued. Overall, the higher response rate for devalued outcomes in the ATD group was not driven by impaired R-O learning, as both groups showed equally good performance in the outcome-devaluation test. Moreover, stronger habitual responding in ATD group was accompanied by a greater decline in plasma tryptophan levels. In contrast, ATD left intact the ability of the subject to use discriminative stimuli to guide instrumental choice at the instrumental learning stage and also to inhibit responding more generally, as reflected in intact performance on the baseline test of response inhibition.

### 5-HT May Modulate the Balance between Appetitive Goal-Directed and Habitual Behavior

Serotonin could potentially modulate the balance between goal-directed and habitual behavioral control at several levels. Thus, depletion of 5-HT neurotransmission has been shown to enhance responsiveness to punishment (Dayan and Huys, 2009; Boureau and Dayan, 2011) whilst disinhibiting previously rewarded but currently punished behavior (Cools et al., 2008).

A failure to update previously positively reinforced associations could arise from cognitive inflexibility, as observed in monkeys after prefrontal serotonin depletion (Clarke et al., 2004). This is also supported by the negative relationship between plasma serotonin level and percentage of responding for the devalued outcomes in the slips-of-action test. The finding is broadly in line with another ATD study that also suggested a facilitatory influence of serotonin on reward processing and choice persistence (Seymour et al., 2012).

### Effects of ATD on Habitual Control Apparently Independent of Response Disinhibition

Alternatively, as serotonin depletion promotes impulsive actions in humans and other animals (Winstanley et al., 2004; Worbe et al., 2014), including deficits in analogous Go/NoGo tasks (Eagle et al., 2008), and disinhibition in Pavlovian aversive conditioning (Crockett et al., 2012a), a greater degree of motor impulsivity in the ATD group could potentially confound our results. However, in this task, the ATD group successfully withheld responding for devalued stimuli in the baseline test, which indicates an intact capacity for action stopping, in line with previous studies that showed no effect of 5-HT depletion on motor impulsivity using stop-signal response inhibition task (Clark et al., 2005; Eagle et al., 2009). Moreover, in both the outcome-devaluation (slips-of-action) and stimulus-devaluation (baseline) tests, the ATD group showed even slower responses for the devalued stimuli compared with BAL, additionally arguing against the hypothesis that greater levels of impulsivity could explain the present results.

### Neural and Neurochemical Substrates

Given the synergistic and opponent relations between the 5-HT and DA system (Winstanley et al., 2005; Boureau and Dayan, 2011; Cools et al., 2011), the change in balance between goal-directed and habitual behavioral control after ATD might result from interactions with the DA system. In rat models, 6-hydroxydopamine-induced lesions of the DA system disrupted habitual responding (Faure et al., 2005), while it was apparently promoted by putatively enhanced DA neurotransmission produced by a sensitising regimen of amphetamine administration (Nelson and Killcross, 2006, 2012). As ATD shifted the behavioral tendency towards habitual responding, this might argue against opponent roles of serotonin and DA in the hypothetical balance between these behavioral control systems, consistent with the findings of de Wit et al. (2012) in female volunteers.

It would be of theoretical as well as clinical value to also test the effects of enhanced 5-HT and DA neurotransmission, for example, as produced by administration of selective serotonin reuptake inhibitors and the DA precursor levodopa on habitual control.

Previous studies in animals and humans have suggested different anatomical substrates for the goal-directed and the S-R habit systems. Thus, the integrity of the sensorimotor striatum, amygdala and infralimbic cortex is required for habitual performance, as shown by experiments in rats with neuronal recording, brain lesions, and pharmacological inactivation (Lingawi and Balleine, 2012; Smith and Graybiel, 2013). Human neuroimaging studies have shown that engagement in goal-directed behavior has been predicted by greater activity of ventromedial prefrontal cortex and its structural connectivity with caudate nucleus (Tanaka et al., 2008; de Wit et al., 2009), whereas greater reliance on habits was associated with activity and structural connectivity of the premotor cortex with the sensorimotor putamen (Tricomi et al., 2009; de Wit et al., 2012a; O’Callaghan et al., 2013). In rodents, overexpression of 5-HT_6_ resulted in reduced habit learning (Eskenazi and Neumaier, 2011) during acquisition of an instrumental learning task. In humans, the expression of 5-HT_6_ receptors has also been linked to cells in the body of the striatum, and to a much lesser degree to the cells of the hippocampus and prefrontal cortex (Marazziti et al., 2012). Disruption of serotonin neurotransmission possibly via 5-HT_6_ receptors in the striatum might thus shift behavioral performance towards a habitual mode. Nonetheless, ATD is not exclusive to a specific 5-HT receptor type, and further pharmacological manipulations with specific 5-HT receptor antagonists, including of the 5-HT_6_ receptor, are required to test this hypothesis. Moreover, further studies using neuroimaging techniques are required to specify the anatomical basis of the effects of tryptophan depletion on 5-HT systems in the brain.

### Study Limitations

First, we did not control for hormonal cycle in female participants. Previous studies showed that hormonal cycle influences reward processing and working memory (Bayer et al., 2013; Hampson and Morley, 2013) and so could potentially have confounded our results, although in fact no interactions were observed with gender, arguing strongly against this possibility.

Second, previous studies have pointed to a lower availability of midbrain serotonin in subjects with family history of major depression (Hsieh et al., 2014). However, previous studies, including our own, have shown that there is no effect of ATD on mood in healthy volunteers (van Donkelaar et al., 2011; Crockett et al., 2012b), so this factor appears unlikely to have influenced the findings.

Third, although we included adequate controls for simple response disinhibitory effects of ATD, it is possible that under certain aversive conditions (Crockett et al., 2012a) or with more difficult control tasks, that disinhibition may have influenced the findings.

Further studies on a larger sample size are needed to confirm the present results, but the present findings were significant even after correcting for multiple comparisons.

## Conclusion

In summary, tryptophan depletion led to a shift from goal-directed to habit-based behavior, hypothetically as a consequence of effects on forebrain 5-HT. These effects might be relevant for understanding certain forms of compulsive behavior occurring in OCD (Cillan and Robbins, 2014), certain eating disorders (Voon et al., 2014), or stimulant drug addiction (Everitt and Robbins, 2005b).

## Statement of Interest

T.W.R. consults for Cambridge Cognition, E. Lilly, Lundbeck, Teva, Shire Pharmaceuticals, and Chempartners and has received research grants from E. Lilly, Lundbeck, and GlaxoSmithKline. E.F.-E. consults for Roche Pharmaceuticals. Y.W. received support from the Fyssen Foundation. G.S. and S.W. have no disclosures.
